# Antibacterial effects of silver–zirconia composite coatings using pulsed laser deposition onto 316L SS for bio implants

**DOI:** 10.1007/s40204-014-0028-5

**Published:** 2014-11-14

**Authors:** G. Pradhaban, Gobi Saravanan Kaliaraj, Vinita Vishwakarma

**Affiliations:** Centre for Nanoscience and Nanotechnology, Sathyabama University, Chennai, 600119 India

**Keywords:** Zirconia, Silver, Biomedical implants, Thin films, PLD, Antibacterial effect

## Abstract

Bacterial invasion on biomedical implants is a challenging task for long-term and permanent implant fixations. Prevention of initial bacterial adherence on metallic implants is an important concern to avoid extracellular matrix (biofilm) secretion from bacteria that is resistant to antibacterial agents. In order to overcome this defect, recently, surface coatings such as zirconia (ZrO_2_) with higher smoothness have been shown to improve implants durability. In the present study, pulsed laser deposition (PLD) was used to deposit ZrO_2_ and silver (Ag)-ZrO_2_ composite coatings onto 316L stainless steel (316L SS). Phase purity, surface roughness and surface morphology, thickness of the coatings and elemental compositions of the coatings were analyzed using X-ray diffraction (XRD), atomic force microscopy (AFM) and scanning electron microscopy (SEM) with energy dispersive X-ray spectroscopy (EDS). Total viable count (TVC) and epifluorescence microscopy analysis were studied to evaluate antimicrobial efficiency of ZrO_2_ and Ag–ZrO_2_ composite coatings using gram negative (gram −ve) *Escherichia coli* (*E.coli*) and gram positive (gram +ve) *Staphylococcus aureus* (*S.aureus*). On the basis of the present study, it could be speculated that ZrO_2_ coatings exhibited antibacterial activity against only *E.coli*, whereas Ag–ZrO_2_ composite coatings showed superior activity against *E.coli* and *S.aureus* strains.

## Introduction

In recent years, the number of implant devices have increased for various implant surgeries takes leading effort across the world (Nathan et al. 2012). The demands of implants have significantly increased not only for new patients but also for patients who must receive revision surgeries. After implantation, the implant materials should not cause allergic and hypersensitive reactions. Hence, biocompatibility is an essential requirement for an artificial implant to exhibit chemical bonding to living tissues upon the formation of bone-like apatite layer on its surface in any simulated body environment (Kokubo 2008; Arisara et al. 2012). In order to improve bioactivity, biocompatibility and implant longevity, thin films which are having nano crystalline achieved considerable breakthrough in the field of biomedical sciences due to their exceptional physical and chemical properties (Holleck 1991). Nanostructured metal oxide thin films and metal complexes have emerged as class materials which are increasingly being studied for health-related applications. Highly ionic metal oxides are interesting in physical, chemical and antibacterial properties (Gu et al. 2007; Jangra et al. 2012).

Among various metal oxides, ZrO_2_ is used extensively for various implant devices because of its good mechanical and biocompatibility properties and used for esthetic devices too. As a ceramic, ZrO_2_ exhibits several advantages such as corrosion resistance, mechanical strength and fracture toughness. The enhanced properties of ZrO_2_ attributed to high crystallinity and nano crystalline ZrO_2_ express better biocompatibility and bioactivity nature (Uchida et al. 2002). The requirement of ZrO_2_ implants have been increased significantly from 4, 28,000 to 2.16 million per year for total knee arthroplasty. Hip replacement is also one of the important applications of ZrO_2_ among the various implants. Chevalior stated that more than 6,00,000 ZrO_2_ femoral have been implanted worldwide (Chevalier 2006). The ceramic ZrO_2_ implant materials, with ISO standard No. 13356, are extensively being used. Even though ZrO_2_ thin films are near ideal biomaterial, their use does not always guarantee adequate results. In this aspect, Microbial invasion and colonization on ZrO_2_ implant materials is still a major challenge against implant durability. Since ZrO_2_ thin film exhibits antibacterial activity against only *E.coli*, it is not active against a wide variety of microbial species and thereby implanted material is prone to bacterial attack that may eventually lead to inflammation and destructive of the implants (Jangra et al. 2012). Among the various bacterial species *Enterococcus faecalis*, *E.Coli*, *Cornybacterium* sp., and *Staphylococcus* sp., predominantly invade the implant materials and cause orthopedic infections (Udo 2009). Hence, microbial invasion is an important crisis for implant success and durability. Diagnosis and treatment for implant failure is the tedious process in case of load bearing and hip, knee artroplasty implants. Recently, addition of antimicrobial metallic materials into metal oxide implants is suitable choice to elevate the antimicrobial property, especially from implant-associated infectious bacteria (Jelinek et al. 2013). In that perspective, Silver has been used in many medical devices such as bone cements, catheters, orthopedic fixation pins, dental implants, and cardiac prostheses (Lansdown 2006). One of the best known uses of silver in medicine is to serve as the primary antimicrobial agent in topical creams to heal severe burns (Lansdown 2007; Atiyeh et al. 2007). Since bacterial infections are associated with prosthetic failures, silver has been considered as a medical coating for broad range of microbial inactivation. Since hydroxyapatite has poor mechanical property, Ag–ZrO_2_ thin composite coatings are studied extensively to enhance biocompatibility, mechanical, and antimicrobial behavior. Ag–ZrO_2_ thin composite coatings have been fabricated using several methods such as sol–gel, physical vapor deposition, and plasma spraying (Ewald et al. 2006; Zheng et al. 2009; Chen et al. 2007). Among the various deposition techniques, pulsed laser deposition (PLD) is one of the novel methods to modify the metal surface. It gives high deposition rate, good stoichiometry, and better adhesion. In this work, ZrO_2_ and Ag–ZrO_2_ composite coatings were prepared by PLD. The structural and surface topography of the coatings were studied by XRD and AFM, respectively. Coating’s thickness and morphology were analyzed by SEM. Elemental composition and mapping were analyzed by EDS. The efficiency of antibacterial properties of ZrO_2_ and Ag–ZrO_2_ composite coatings were evaluated and compared against *E.coli* and *S.aureus* bacteria.

## Methods

### Substrate pretreatment

Medical grade stainless steel 316L (316L SS) and silicon substrate (for characterization) were selected as a substrate and pretreatment was performed. Briefly, substrates were cut into 1 × 1 cm^2^ using automated cutting machine (Struers, Tegramin-25, Denmark). Mirror polishing was done using automated polishing machine (Struers A/S, DK-2750 Ballerup, Denmark) and silicon carbide emery papers (240–1000 mesh sizes) were used to reduce the surface roughness of the substrates. Finally, mirror polished substrates were treated with trichloroethylene in order to remove the rust and ultrasonicated for 15 min before coatings.

### Deposition of silver-ZrO_2_ composite coatings and their characterizations

**Fig. 1:**
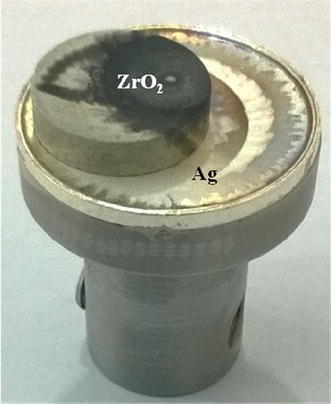
Target holder consists of Ag and ZrO_2_

The commercial ZrO_2_ powders with 99.99 % purity were pressed with 40 MPa in the 8 mm mold to prepare disc. This disc was subjected to heat treatment at 900 °C for 5 h in air atmosphere with heating rate of 10 °C/min. The resultant 8 mm diameter and 3 mm thickness ZrO_2_ disc and commercial silver with 25 mm diameter and 2 mm thickness disc were used as targets in this experiment. The vacuum chamber was evacuated by air cooled turbo-molecular pump and kept at 1 × 10^−6^ m bar as a base pressure as well as deposition pressure. A Q-switched Nd-YAG laser operating at wavelength 355 nm with fluence of 2 J/cm^2^ and repetition rate of 10 Hz is allowed into the chamber through a quartz window. The laser beam with 2 mm diameter is focused on the targets at the angle of 45^°^. For Ag–ZrO_2_ composites coatings, same 8 mm diameter ZrO_2_ target is fixed on 25 mm Ag target (Fig. 1). Target and substrate distances were kept at 45 mm. Prior to deposition, both targets were ablated with 3,000 pulses to remove the surface contamination to get high-purity coatings with shutter between targets and substrates and 45,000 pulses have been applied on target for each coating. The substrates were kept at a constant temperature of 200 °C with ramping rate of 10 °C/min for both the deposition. Both depositions are performed at similar conditions.

The phase structure of ZrO_2_ and Ag–ZrO_2_ composite coatings were analyzed by glancing angle X-ray diffractometer (Rigaku, smart lab 9 KW, japan) using a CuK*α* = 1.5406Å radiation source. The surface of the coating was characterized by molecular imaging atomic force microscopy (AFM) using SLOVER PRO, multi mode scanning probe microscope manufactured by M/s NTMDT, the Irelands.

## Bacterial adhesion study

### Bacterial strains

*E.coli* (MTCC 443) and *S.aureus* (MTCC 3160) bacterial strains were used for this study. Cultures were maintained on nutrient broth at 37 °C.

### Preparation and inoculation of organisms

The bacterial suspensions were prepared by taking a single colony from the stock bacterial culture with a loop and inoculating 20 ml of sterile nutrient broth in a 100-ml Erlenmeyer flask. The flask was then incubated in a shaking incubator at 37 °C at 110 rpm for 12 ± 2 h. After incubation, 0.4 ml of the overnight inoculum was transferred to a 100-ml Erlenmeyer flask containing 20 ml nutrient broth and incubated in a shaking incubator for 3 h at 37 °C and 110 rpm. The number of bacteria in the 3 h culture was estimated by measuring the optical density of the culture at 660 nm (Corning colorimeter 253). An optical density of between 0.1 and 0.3 was roughly equal to a concentration 2.5 × 10^8^ CFU/ml.

### Sterilizations of test samples

ZrO_2_ and Ag–ZrO_2_ composite onto 316L SS were sterilized under UV lamp with 5 cm distance for 10 min to obtain surface sterilization. Post exposure, specimens were transferred to sterilized disposable petriplates to avoid cross contamination. All the specimens were retained in the petriplates during each experiment.

### Antibacterial analysis

The cultured bacterial strains were inoculated in 10 ml phosfate-buffered saline (PBS; g/l: KCl 0.2, KH_2_PO_4_ 0.2, NaCl 8.0, Na_2_HPO_4·12_H_2_O) solution to reach approximate target concentration of 1 × 10^5^ colony forming units/milliliter (CFU/ml). The saline solution containing bacteria was used for the ‘drop-method’ antibacterial experiments (Sun et al. 2008). The samples were placed in the sterilized petridishes. Then 100 μl of saline solution containing bacteria was added dropwise onto the surface of each deposited film. The samples were laid at ambient temperature for 12 h to evaluate absolute initial antimicrobial properties of test specimens. Incubated samples were washed with 5 ml of PBS in the sterilized petridish. Then 10 μl each of bacteria suspension was dispersed on the culture medium and incubated for 24 h at 37 °C. After that, Total viable count (TVC) was enumerated to evaluate viable bacterial cells arising from solid medium. The number of bacteria-forming units (CFU) was counted, and the reduction of bacteria, *R*, was calculated from

1 $$R=\frac{\left(B-A\right)}{B}100\;\left(\%\right),$$

where *A* is the CFU recovered from the uncoated 316L SS (data is not shown) and *B* is the CFU recovered from the coated samples over the desired contact period (24 h).

For live/dead cell analysis, same quantity of bacterial suspension was poured onto test specimens for 30 min. The incubated samples were gently washed in PBS to remove the unbound bacterial cells. Finally, acridine orange (AO) was added onto specimens and observed using Nikon Eclipse E600 Epifluorescence microscope (excitation filter BP 490; barrier filter O515).

## Results and discussion

**Fig. 2:**
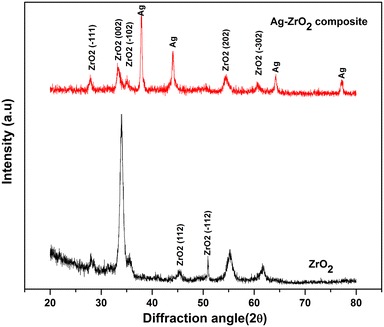
XRD pattern of ZrO_2_ and Ag–ZrO_2_ composite coatings

Figure 2 shows X-ray diffraction patterns of ZrO_2_ and Ag–ZrO_2_ composite coatings deposited at 200 °C onto 316L SS substrate with parallel-beam (PB) diffraction geometry. ZrO_2_ coating shows the diffraction at (002), (102), (112), (221), (202) and (302) with corresponding 28.15, 33.92, 35.56, 45.32, 50.87, 55.19 and 61.66° 2θ positions. Ag–ZrO_2_ composite coatings can also be identified with (111), (002), (−102), (112), (−221), (202) and (302) planes with corresponding 2θ position of 27.95, 33.30, 35.15, 37.93, 44.09, 54.47, 60.74, 64.24 and 77.19°. For the estimation of average crystalline size, the following Scherrer equation is employed (Suryanarayan and Norton 1998).

2 t=0.9λ/βcosθ

where *λ* = wavelength of X-ray source, *β* = full width half maximum (FWHM) corresponding to XRD peaks. 

The crystalline size of ZrO_2_ is calculated from its peak broadening and crystalline size found to be 16.19 nm. In case of Ag–ZrO2 composite coatings, crystalline size of ZrO_2_ found to be 13.56 nm. The crystalline of Ag was calculated as 27.14 nm and no secondary phase of Ag was observed. It clearly indicates that crystallinity of Ag fully improved and crystalline size was larger than that of ZrO_2_. At this low substrate temperature (200 °C), some grains of the ZrO_2_ material grows amorphous nature, whereas Ag grain growth fairly formed crystalline nature and this formation inhibited further grain growth of ZrO_2_ in Ag–ZrO_2_ composite coatings.

**Fig. 3:**
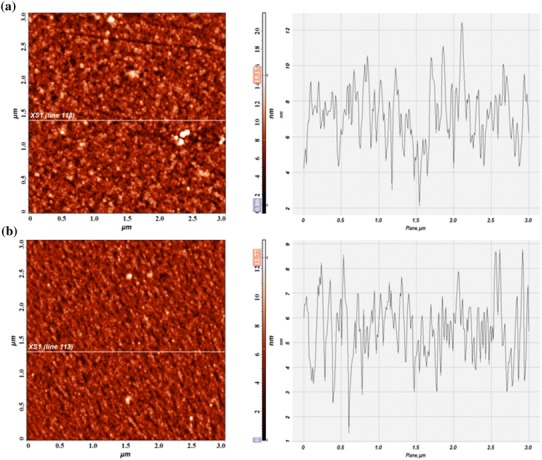
Two-dimensional (2D) AFM images and surface profiles of ZrO_2_ (**a**) and Ag–ZrO_2_ composite coatings (**b**) coated 316L SS from approximately 3 × 3 μm scanned areas

**Fig. 4:**
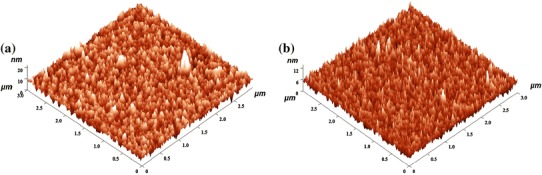
Three-dimensional (3D) projections of typical AFM images and surface profiles of ZrO_2_ (**a**) and Ag–ZrO_2_ composite coatings (**b**) coated 316L SS from approximately 3 × 3 μm scanned areas

Typical nano topography using two-dimensional (2D) and three-dimensional (3D) images of AFM with surface profiles of ZrO_2_ and silver-ZrO_2_ composite coatings are shown in Figs. 3, 4. A comparative AFM analysis indicated that the slightly heterogeneous surface with increased grains exhibited in ZrO_2_ coating; for Ag–ZrO_2_ composite coatings, homogenous surfaces with fine topographical alterations are observed. The degree of roughness and grains in ZrO_2_ coating (*R*_a_ = 1.4541 nm) is slightly increased than silver-ZrO_2_ (*R*_a_ = 1.04816 nm) coatings. ZrO_2_ coatings showed the maximum and minimum grain sizes in the range of 4–10 nm along with few agglomerations. For Ag–ZrO_2_ composite, it has shown as 3–8 nm. From 2D and 3D profile of AFM surface profile analysis concludes that the addition of silver content in ZrO_2_ results in a minute decrease of both the grain size and surface roughness due the nucleation matter (Subramanian and Jayachandran 2007).

**Fig. 5:**
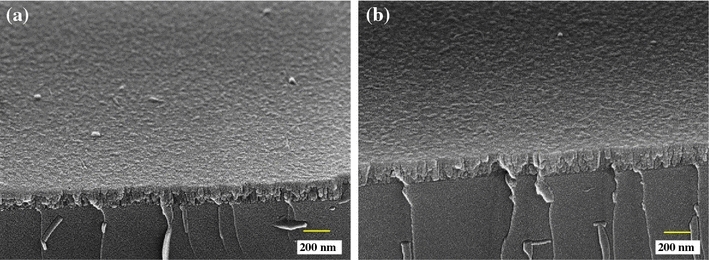
Cross-sectional images of ZrO_2_ and Ag–ZrO_2_ composite coatings

Morphology as well as cross section of the ZrO_2_ and Ag–ZrO_2_ composite coatings is shown in Fig. 5. Both ZrO_2_ (Fig. 5a) and Ag–ZrO_2_ (Fig. 5b) composite coatings showed dense, smooth and defect-free surface along with smaller grain size. Few droplets or agglomeration of grains was also observed in both coatings. Formation of droplets is one of the intrinsic properties of laser ablation technique (Willmott and Huber 2000). The total thickness of the ZrO_2_ and Ag –ZrO_2_ composite coatings is evaluated from the cross-sectional view of the samples and found to be 120 and 154 nm, respectively. The slight variation of thickness attributed to the difference in ablation rate of Ag and ZrO_2_. Generally, pure metals have more ablation rates than ceramic materials in PLD technique. In this case the ablation rate of metallic Ag is comparatively more than that of ZrO_2_ ceramic material.

**Fig. 6:**
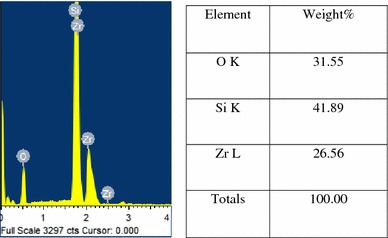
Compositional analysis of ZrO_2_ coating

**Fig. 7:**
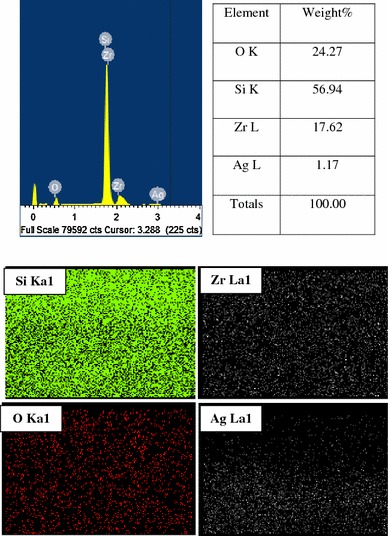
Elemental and compositional analysis of Ag–ZrO_2_ composite coating

Figure 6 shows the compositional analysis of ZrO_2_ coatings contains both zirconium (Zr), oxygen (O) and silicon (Si). Figure 7 shows the compositional analysis and elemental mapping of Ag–ZrO_2_ composite coatings, in which, the wt % of Ag was 1.17. Si peak was also observed due to the surface effect. From the compositional analysis, the presence of Zr, O and Ag, Zr, O were confirmed in ZrO_2_ and Ag–ZrO_2_ composite coatings.

Antimicrobial activity of ZrO_2_ and Ag–ZrO_2_ composite coatings coated 316L SS was evaluated by determining TVC. ZrO_2_ coating shows antibacterial activity of about 99 % (data are not shown) against *E.coli* and exhibits very less antibacterial efficiency about 46 % against *S.aureus.* On the other hand, drastic growth arrest is observed on Ag–ZrO_2_ composite coatings with the antimicrobial efficiency of 99.5 and 99 % for *E.coli* and *S.aureus*, respectively. During incubation, when aqueous bacterial solutions are exposed onto coating surfaces, many of the bacteria have a possibility to attach directly to the surface. Since silver is present on the surface of the samples, metallic silver ions leached from coating surface and affect both adhered and non adhered bacterial cell wall (Sondi and Sondi 2004). The mechanism of both bacterial strains against Ag–ZrO_2_ composite coatings varies as (a) Ag ions may bind with bacterial cell surface proteins such as porins, OmpF and OmpC and produce imbalanced membrane diffusion in gram-negative bacteria; (b) metallic Ag ions bind with surface anionic proteins and lost their membrane rigidity and increase the cell wall porosity. Hence, the above mechanisms elevate oxidative stress; thereby free radicals production occurs which arises reactive oxygen species, resulting structural changes and cell damage occur. In addition to that, the metallic silver ions are effectively involved in binding with sulfur or phosphorus-containing soft bases, such as R-S-R, R-SH, RS^−^ or PR_3_ which is present in cell membrane and phosphate group of DNA which is located inside the cell (Bragg and Rainnie 1974; McDonnell and Russell 1999; Darouiche 1999). As a result, the bacterial growth is effectively inhibited within a short time of exposure. From the TVC results, it could be speculated that ZrO_2_ coating worked out exhibited better antibacterial activity only against *E.coli* and no activity for *S.aureus*. It is due to the heterogeneity structure and chemical composition of the outer membrane of gram +ve and gram −ve bacteria (Thomas et al. 2010). The calculated TVC of gram (−)ve and gram (+)ve test organisms are to be 1.78 × 10^2^, 1.02 × 10^4^ for ZrO_2_ coating and 92, 164 for Ag–ZrO_2_ composite coatings, respectively.

**Fig. 8:**
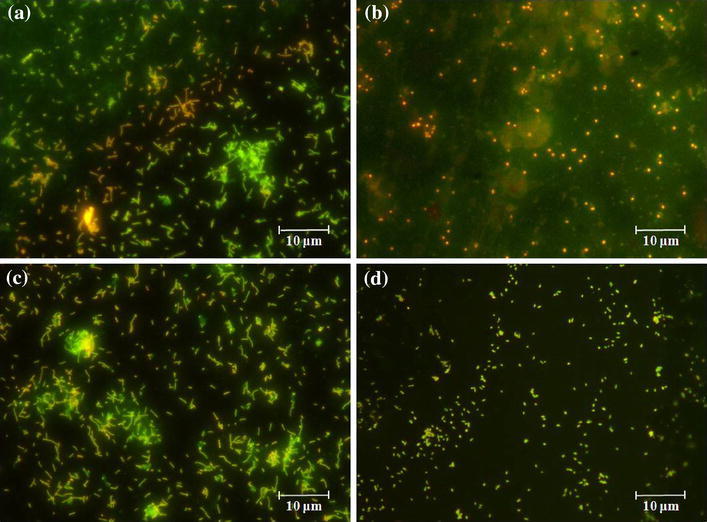
Epifluorescence microscopy images of ZrO_2_ (Fig. 5**a***E.Coli* and **b***S.aureus*) and Ag–ZrO_2_ composite coatings (Fig. 5**c***E.Coli* and **d***S.aureus*)

Figure 8 depicts epifluorescence microscopy images of ZrO_2_ and Ag–ZrO_2_ composite coated 316L SS. In principle, live cells exhibited orange fluorescence and dead cells exhibited green color due to the interaction of AO with cellular RNA (Vinita Vishwakarma et al. 2009). Epifluorescence results exhibited similar results with TVC analysis. Bacterial inoculated ZrO_2_ coating shows green fluorescence for *E.coli* bacteria; orange color fluorescence for *S.aureus*. Result concludes that ZrO_2_ coating shows bacterial activity against *E.coli* and fails to express the bactericidal nature against *S.aureus*. In case of Ag–ZrO_2_ composite coatings, both *E.coli* and *S.aureus* show green fluorescence indicates dead cells. The exposed bacteria are interacting with Ag ions which undergo severe changes in and around the bacterial cell wall thereby elevating deterioration of bacterial cell wall. Besides, strong interaction of silver ions with intracellular components affects further cellular RNA synthesis which leads bacterial death.

## Conclusions

ZrO_2_ and Ag–ZrO_2_ composite coatings were successfully prepared using PLD technique. XRD examination reveals that monoclinic phase for ZrO_2_ and mixed phase for Ag–ZrO_2_ composite coatings were obtained. AFM morphology of the coatings clearly indicated that the grains are evenly distributed. Elemental analysis by EDS confirmed the presence of Ag in Ag–ZrO_2_ composite coatings about 1.17 wt % of Ag. The antimicrobial efficiency was performed using TVC and epifluorescence microscopy analysis in which Ag–ZrO_2_ composite coatings shows superior antibacterial when compare to ZrO_2_ coating. Hence, the 1.17 wt % Ag content exhibits superior antibacterial activity and it may be the good candidate for biomedical applications.
